# Draft Genome Sequence of *Cutaneotrichosporon curvatus* DSM 101032 (Formerly *Cryptococcus curvatus*), an Oleaginous Yeast Producing Polyunsaturated Fatty Acids

**DOI:** 10.1128/genomeA.00362-16

**Published:** 2016-05-12

**Authors:** Thomas Hofmeyer, Silke Hackenschmidt, Florian Nadler, Andrea Thürmer, Rolf Daniel, Johannes Kabisch

**Affiliations:** a Junior Research Group Drop-In Biofuels, Ernst-Moritz-Arndt-Universität, Greifswald, Germany; b Göttingen Genomics Laboratory, Göttingen, Germany

## Abstract

*Cutaneotrichosporon curvatus* DSM 101032 is an oleaginous yeast that can be isolated from various habitats and is capable of producing substantial amounts of polyunsaturated fatty acids. Here, we present the first draft genome sequence of any *C. curvatus* species.

## GENOME ANNOUNCEMENT

*Cutaneotrichosporon curvatus*, until recently known as *Cryptococcus curvatus*, is an oleaginous yeast of great interest for biotechnological applications due to the increasing demand of microbial lipids as a feedstock for the production of sustainable and renewable fuels and fine chemicals (1, 2). The fungus is widespread and can be isolated, for example, from foodstuff, like raw milk and lettuce, or from marine sediments (3–5).

*C. curvatus* has the capability to utilize a broad spectrum of different renewable carbon sources, like glucose, sucrose, lactose, xylose, glycerol, arabinose, and cellobiose (6). Furthermore, it was shown to be capable of utilizing these and other substrates, like pretreatment-derived hemicellulose, organic waste from the food industry, or active sludge to produce lipids in numerous batch and fed-batch cultivations (7–10). In this context, the *C. curvatus* genome sequencing revealed several hits of coding sequences for enzymes required for the degradation of renewable polymers, such as cellulose (e.g., putative cellulases, Ccurv_10480 and Ccurv_12500) and chitin (putative chitinase, Ccurv_32040), as well as enzymes for the metabolism of the resulting monomers.

Under nitrogen-limiting conditions, *C. curvatus* can accumulate lipids up to 60% of the dry cell weight (11). The fatty acid profile of this study’s strain contains about 50% unsaturated fatty acids, among them α-linolenic acid (C_18:3_, *n*-3); hence, *C. curvatus* can be denoted as a natural omega-3 fatty acid producer (data not shown). However, no genome sequence has been publicly available. Here, we report the draft genome sequence of *C. curvatus* DSM 101032, alongside a JBrowse (12)-based genome browser of the functionally annotated genome, which is available at http://genomes.chemie.uni-greifswald.de/.

The analyzed strain, a progeny of *C. curvatus* PYCC 2565, is deposited at DSMZ as DSM 101032. The genomic DNA of *C. curvatus* was prepared according to the rapid preparation of yeast DNA protocol (13, 14) and treated with RNase (NEB), according to the manufacturer’s instructions. Strain taxonomy was confirmed by amplification and sequencing of internal transcribed spacer (ITS) DNA with the ITS1 and ITS4 primers (15).

Shotgun libraries were generated using the Nextera XT DNA sample preparation kit, according to the manufacturer’s instructions. The whole genome of *C. curvatus* was sequenced with the Genome Analyzer IIx (Illumina, San Diego, CA) by the Göttingen Genomics Laboratory (G2L [http://appmibio.uni-goettingen.de/index.php?sec=g2l]). The libraries were sequenced in a 112-bp paired-end single-indexed run. The resulting 19.9 × 10^6^ paired-end reads were assembled using SPAdes 3.5.0, yielding 366 large contigs (≥10,000 bp), an *N*_50_ contig size of 59,188 bp, and a total length of approximately 18.28 Mbp, with a G+C content of 59.41% (16). Open reading frames were determined using AUGUSTUS 2.7, with the species set to *Cryptococcus neoformans* (17).

The draft genome sequence presented notable progress in the further understanding of *C. curvatus* and will lead to the development of useful biomolecular tools and protocols (18). These data will indubitably enable a better exploitation of *C. curvatus* metabolism and open up new avenues on the road to a renewable production of single-cell oil.

### Nucleotide sequence accession numbers.

This whole-genome shotgun project has been deposited at DDBJ/EMBL/GenBank under the accession no. LDEP00000000. The version described in this paper is version LDEP01000000.
